# Supplementary material for: BEL Commons: an environment for exploration and analysis of networks encoded in Biological Expression Language
Source: Database (Oxford). 2018 Dec 14;2018:bay126. doi: 10.1093/database/bay126 (PMC6301338; doi:10.1093/database/bay126)
Supplement: Supplementary Data [file bay126_supp.pdf]

BEL Commons: Supplementary Information

Supplement Overview

- **An Overview of Web Services:** A summary of the web applications that handle biological networks.
- **BEL Commons Implementation Details:** Technical details about the architecture and the technologies used to implement BEL Commons.
- **Tutorial:** References to the tutorial that outlines the Application Scenario presented in this paper.
- **References**: References used in this document

An Overview of Web Applications

Here, we present a brief overview on some of the web applications mentioned in the introduction of the manuscript (**Supplementary Table 1**). We also present a comparison of features (**Supplementary Table 2**) and visualization techniques (**Supplementary Table 3**) implemented by these applications.

Platform	Focus	Export	Visualization
WikiPathways (Pico *et al.*, 2008)	Crowdsourcing biological model building	SBML	PathVisio, Cytoscape
Pathway Commons (Cerami *et al.*, 2010)	Aggregating pathway databases BioPax, SMBL, SIF, etc.	BioPAX	CyPath2, PaxToolsR, ChiBE
NDEx (Pratt *et al.*, 2015)	General network storage from CX	CX	Cytoscape.js
GraphSpace (Braradwaj *et al.*, 2017)	General network storage from Cytoscape	Cytoscape JSON	Cytoscape.js
SBV Improver (Meyer *et al.*, 2015)	Verification of semi-automated relation extraction results	SIF, JGIF	Custom Viewer
Causal Biological Networks Database (Boué *et al.*, 2015)	BEL network storage	SIF, JGIF	Custom Viewer
NeuroMMSig (Domingo-Fernández *et al.*, 2017)	Enrichment analysis	BEL, JSON	Custom Viewer

Supplementary Table 1. A summary of several systems and networks biology web applications

WikiPathways is a repository of pathways that is built and maintained using crowdsourcing (Pico *et al.*, 2008). It uses the Systems Biology Markup Language (SBML) format to store its networks and has deep semantic integration with external databases.

Pathway Commons is an aggregation of a significant number of publicly available pathway databases accessible in the BioPAX format and through a web interface (Cerami *et al*., 2010).

The **Network Data Exchange** (NDEx) is a repository where scientists can upload, share, and distribute networks with fine-granular user rights management (Pratt *et al*., 2015). It accepts many formats (BioPAX, BEL, simple interchange format, etc.) and uses CX as a latent storage medium. It has a rudimentary network query systems (e.g. search for nodes and their neighborhoods) and tight integration with Cytoscape to fill in missing features in network analysis and to provide in-browser visualization with high customization.

GraphSpace is a service for uploading, sharing, and viewing networks in the Cytoscape format (Braradwaj *et al*., 2017). While it has the benefit of being domain-agnostic, it has begun to identify and address the issue of layouts in visualization of biological networks.

The **Causal Biological Networks Database** is a repository for manually curated biological knowledge assemblies encoded in Biological Expression Language (BEL) (Boué *et al*., 2015). It provides a BEL-specific viewer and the ability to export in simple interchange format (SIF) and JSON graph interchange format (JGIF) for use in general network visualization programs. Additionally, its underlying data is partially dependent on the **SBV Improver**, which extends current knowledge assemblies using crowdsourcing in a semi-automated text mining pipeline for relation extraction from biological literature (Meyer *et al*., 2015).

NeuroMMSig is a repository of manually curated biological knowledge in the domain of neurodegenerative disease that focuses on the application of a mechanism enrichment algorithm to identify patient subgroups in clinical data sets (Domingo-Fernández *et al*., 2017). It also provides data integration of clinical and molecular data and a viewer for qualitative observation. Topological analysis, enrichment analysis.

**Application**	**Create**	**Edit**	**Integrate**	**Share**	**Visualize**	**Data-Driven Analysis**
BELIEF (Madan *et al*., 2016)	✔	✔				
WikiPathways	✔	✔		✔	✔	
SBV Improver		✔			✔	
Pathway Commons			✔		✔	
NDEx				✔	✔	
GraphSpace				✔	✔	
NeuroMMSig					✔	✔

Supplementary Table 2. A summary of the features of several systems and networks biology web applications

Visualization Tool	Summary
Cytoscape (Shannon *et al.*, 2003)	A Java-based desktop client for general network visualization and analysis
Cytoscape.js (Franz *et al.*, 2015)	An in-browser Javascript library for creating network visualization
PathVisio (Kutmon *et al.*, 2015)	Biological pathway drawing and analysis tool
D3.js	An in-browser Javascript library for creating a variety of visualization
CellDesigner (Matsuoka *et al.*, 2014)	A biologically-oriented editor for gene-regulatory and biochemical networks

Supplementary Table 3: A summary of visualization tools for systems and networks biology

BEL Commons Implementation Details

BEL Commons follows a model-view-controller (MVC) software architecture. Its back-end is implemented in the Python programming language using the Flask microframework (http://flask.pocoo.org) and a suite of its extensions (**Supplementary Table 6**). Its core functionalities are implemented with PyBEL (Hoyt *et al.,* 2018) and exposed with Flask via a RESTful API documented with an OpenAPI specification (https://www.openapis.org). This API implements portions of the OpenBEL API specification to allow for interoperability with legacy BEL applications.

BEL Commons needs to be able to concurrently handle multiple expensive operations such as parsing, validation, compilation, and analysis. Because Python is inherently single-threaded, we have used the RabbitMQ (https://www.rabbitmq.com) message queue and celery (http://www.celeryproject.org) to implement asynchronous operations. The web application is served using uWSGI (**Supplementary Table 4**).

Its analytical service (i.e. the heat diffusion workflow) relies heavily on the scientific Python stack, including NumPy (Travis and Oliphant, 2006), Pandas (McKinney, 2010), and Scikit-learn (Pedregosa, *et al*., 2012) to take advantage of their fast implementations of matrices and mathematical operations in C, C++, and Fortran. Network algorithms, including attribute selection and path finding algorithms, were all implemented using NetworkX (Hagberg et al., 2008) and derivative classes of the `networkx.MultiDiGraph` class in PyBEL (**Supplementary Table 5**).

The front-end is implemented with jQuery (https://jquery.com), D3.js (https://d3js.org), and Bootstrap (https://getbootstrap.com) along with other Javascript libraries (**Supplementary Table 7**). BEL Commons uses MySQL (https://www.mysql.com) as a relational database management system and SQLAlchemy (https://www.sqlalchemy.org) as an object relational mapper to easily extend the SQL network data models provided by PyBEL and to create new data models for users, projects, and analyses.

Technology	Functionality
MySQL	Relational database management system
RabbitMQ	Message queue
Celery	Enables asynchronous jobs
uWSGI	WSGI Web Server

Supplementary Table 4: A summary of back-end technologies used by BEL Commons

Package	Functionality
NetworkX	Network data structure
PyBEL	Parsing, compilation, storage, and conversion of BEL graphs
PyBEL-Tools	Summarizing, querying, and analysis of BEL graphs
SQLAlchemy	Object-relational Mapper
Flask	Microframework for web applications
NumPy	Multi-dimensional array data structure and mathematical operations
Pandas	Data frames and data structures
Scikit-learn	Machine learning

Supplementary Table 5: A summary of Python packages used by BEL Commons

Flask Package	Functionality
Flask	An integrated web server and template manager that wraps many of the low level functions in an easy-to-manage programming interface
Flask-Security	Integrated login and role management system
Flask-Admin	Provides an interface for users to input data such as the project editing form.
Flask-WTF	Underpins the generation and processing of forms
Flask-Bootstrap	Templating for using standard front-end assets like Bootstrap and jQuery
flasgger	Produces Swagger specification and Swagger UI page automatically to document the RESTful API.

Supplementary Table 6: As an addendum to **Supplementary Table 5**; a summary of extensions to the Flask Python package used by BEL Commons

Javascript Library	Functionality
jQuery	Provides manipulation of DOM, CSS, and general-purpose javascript
D3.js	DOM manipulation and network visualization
C3.js	Generate charts
ParCoords.js	Generates parallel coordinates charts
D3-context-menu	Additional user interactions with network visualization
Select2	User interface for autocompletion and selecting lists
InspireTree	Builds tree for annotation browser
Bootstrap Toggle	User interface for slider buttons

Supplementary Table 7: A summary of front-end Javascript libraries used by BEL Commons

Tutorial

A tutorial and accompanying data to reproduce the analysis presented in **Figure 5** of the manuscript can be found on GitHub at https://github.com/cthoyt/bel-commons-manuscript.

References

Bharadwaj, A., Singh, P., Ritz, A., *et al.* (2017). GraphSpace: stimulating interdisciplinary collaborations in network biology. *Bioinformatics*, *33*(19), 3134-3136.

Boué, S., Talikka, M., Westra, J., *et al.* (2015). Causal biological network database: a comprehensive platform of causal biological network models focused on the pulmonary and vascular systems. *Database*, *2015*, bav030.

Cerami, E. G., Gross, B. E., Demir, E., *et al.* (2010). Pathway Commons, a web resource for biological pathway data. *Nucleic acids research*, *39*(suppl_1), D685-D690.

Domingo-Fernández, D., Kodamullil, A., Iyappan, A., et al. (2017). Multimodal Mechanistic Signatures for Neurodegenerative Diseases (NeuroMMSig): a web server for mechanism enrichment. Bioinformatics, 33(22), 3679-3681.

Franz, M., Lopes, C. T., Huck, G., *et al*. (2015). Cytoscape.js: A graph theory library for visualisation and analysis. Bioinformatics, 32(2), 309–311.

Frère, S. G. a, & Lüthi, A. (2004). Pacemaker channels in mouse thalamocortical neurons are regulated by distinct pathways of cAMP synthesis. The Journal of Physiology, 554(Pt 1), 111–125.

Finn, R. D., Attwood, T. K., Babbitt, P. C., et al. (2016). InterPro in 2017—beyond protein family and domain annotations. Nucleic acids research, 45(D1), D190-D199.

Finn, R. D., Coggill, P., Eberhardt, R. Y., et al. (2015). The Pfam protein families database: towards a more sustainable future. Nucleic acids research, 44(D1), D279-D285.

Hagberg, A. A., Schult, D. A., & Swart, P. J. (2008). Exploring network structure, dynamics, and function using NetworkX. Proceedings of the 7th Python in Science Conference (SciPy 2008), (SciPy), 11–15.

Kutmon, M., van Iersel, M. P., Bohler, A., *et al*. (2015). PathVisio 3: an extendable pathway analysis toolbox. *PLoS Computational Biology*, *11*(2), e1004085.

Madan, S., Hodapp, S., Senger, P., *et al*. (2016). The BEL information extraction workflow (BELIEF): Evaluation in the BioCreative V BEL and IAT track. *Database*, *2016*(September 2017), 1–17.

Massone, S., Vassallo, I., Fiorino, G., et al. (2011). 17A, a novel non-coding RNA, regulates GABA B alternative splicing and signaling in response to inflammatory stimuli and in Alzheimer disease. Neurobiology of Disease, 41(2), 308–317.

Matsuoka, Y., Funahashi, A., Ghosh, S., & Kitano, H. (2014). Modeling and simulation using CellDesigner. *Methods in Molecular Biology (Clifton, N.J.)*, *1164*, 121–45.

McKinney, Wes. Data Structures for Statistical Computing in Python, Proceedings of the 9th Python in Science Conference, 51-56 (2010)

Meyer, P., Hoeng, J., Rice, J. J., *et al*. (2012). Industrial methodology for process verification in research (IMPROVER): toward systems biology verification. *Bioinformatics*, *28*(9), 1193-1201.

Palmer, A., Verbitsky, M., Suresh, R., *et al.* (2005). Gene expression differences in mice divergently selected for methamphetamine sensitivity. Mammalian Genome : Official Journal of the International Mammalian Genome Society, 16(5), 291–305.

Pedregosa, F., *et al*. (2012). Scikit-learn: Machine Learning in Python. *Journal of Machine Learning Research*, *12*, 2825–2830.

Pico, A. R., Varoquaux, G., Gramfort, A., *et al*. (2008). WikiPathways: pathway editing for the people. *PLoS biology*, *6*(7), e184.

Pierson, R. C., Lyons, A. M., & Greenfield, L. J. (2005). Gonadal steroids regulate GABAA receptor subunit mRNA expression in NT2-N neurons. Brain Research. Molecular Brain Research, 138(2), 105–15.

Pratt, D., Chen, J., Welker, D., *et al.* (2015). NDEx, the network data exchange. *Cell systems*, *1*(4), 302-305.

Shannon, P., Markiel, A., Owen O., *et al*. (2003). Cytoscape: a software environment for integrated models of biomolecular interaction networks. *Genome Research*, (13), 2498–2504.

Travis E, Oliphant. A guide to NumPy, USA: Trelgol Publishing, (2006).
